# Comparison of Different Finishing and Polishing Systems on Surface Roughness and Bacterial Adhesion of Resin Composite

**DOI:** 10.3390/ma15217415

**Published:** 2022-10-22

**Authors:** Yoav Pietrokovski, Dan Zeituni, Adi Schwartz, Nurit Beyth

**Affiliations:** Department of Prosthodontics, Hadassah Medical Center, Faculty of Dental Medicine, Hebrew University of Jerusalem, Jerusalem 9112102, Israel

**Keywords:** resin composite, esthetics, finish and polishing, adhesive dentistry, minimally invasive dentistry

## Abstract

Insufficient dental restoration finishing and polishing may lead to plaque accumulation, gingival inflammation, staining, caries, and esthetic impairment. Here, the effect of two finishing and polishing systems on surface roughness and bacterial adhesion were evaluated. Two finishing and polishing kits were evaluated: diamond burs (Shine 1-2, Strauss & Co, Raanana, Israel) and paper discs (Sof-Lex 3M ESPE, Seefeld, Germany) (*n* = 30 each). For each group surface roughness was evaluated using an optical profilometer (Contour GT-K1, Bruker, Billerica, MA, USA) (*n* = 10). Surface bacteria were evaluated for biofilm biomass using crystal violet (CV) staining (absorbance measured at 538 nm) and viable counts (CFU/mL) (*n* = 20). The control group included polymerized discs against a Mylar strip (*n* = 30). Student’s *t* test and one-way ANOVA were used for statistical evaluation. Diamond burs, paper discs, and control average surface RA were 169.4 ± 45.2 µ, 364 ± 77.7 µ, and 121.2 ± 18.1 µ, respectively. There was a significant difference found between all groups (*p* < 0.00001). Bacterial biomass on diamond burs, paper discs, and control samples were 0.458 ± 0.161, 0.507 ± 0.139, and 0.446 ± 0.142, respectively (*p* = 0.257). Viable bacterial counts (CFU/mL) on diamond burs, paper discs, and control samples were 2.25 × 10^4^, 2.95 × 10^4^, and 2.75 × 10^4^, respectively (*p* = 0.856). A comparison between two finishing and polishing kits showed that the shine 1–2 diamond bur kit produced a smoother surface than the polishing disc kit. No differences were found in the biofilm biomass quantification and bacterial viable count between the groups.

## 1. Introduction

Proper finishing and polishing of dental restorations are critical features that improve esthetics and prolongs the restorations longevity. Rough surfaced restorations may lead to plaque accumulation, gingival inflammation, marginal staining, caries, and esthetic impairment [1,2,3]. Oral conditions offer difficult surroundings for survival of the dental restorations. Various factors may affect the surface roughness and the adherence of the bacterial plaque, such as the type of polymerization, parafunction, and the patient’s diet [4,5].

Current trends in modern dentistry show an increasing shift towards the use of resin composite materials as plastic restorations, instead of amalgam [6]. Due to their aesthetic properties, dental resin composite materials are being widely employed [7]. In addition to the tooth color advantage, resin composites have good physical, mechanical, chemical, optical, thermal, and wear properties [8]. The two major components of resin composite materials are the resin matrix (organic) and ceramic (inorganic) fillers. The resin matrix’s main elements are monomers, diluents, photo initiators, accelerators, and coupling agents [9]. In recent years, nano particles and nano fibers have been employed as fillers due to their excellent aesthetic, bioactivity, and biocompatibility properties [7].

The ability to achieve a highly polished surface in resin composite restorations is affected by several factors such as filler/matrix ratio, the size of the filler particles, and the means of finishing and polishing the restoration.

Secondary caries and gingival inflammation caused by a poorly finished composite restoration may lead to failure of the restoration, enamel and dentin destruction, loss of periodontal attachment, pain, and eventually loss of the tooth [10,11,12]. Hence, there is an importance of finding an efficient and economic technique to achieve a highly polished composite restoration.

The final steps in the creation of a dental composite restoration are finishing and polishing. Finishing is defined as the refinement of form and is done prior to polishing [13], which is the final step taken in order to make the restoration’s surface smooth and glossy by friction and giving it luster [13].

Most of the methods to reach a desirable resin composite restoration surface involve using frictional agents applied sequentially in progressively finer grits of an abrasive medium [14]. The final result depends on various factors such as particle size, filler/resin ratio, and polishing materials. The different components of composite materials respond differently to abrasion [15] and challenge the clinician to achieve a desirable high-polished surface. The wide variety of finishing and polishing systems consists of aluminum-oxide-coated abrasive paper discs, silicone discs, tungsten carbide finishing burs, abrasive impregnated rubber cups, abrasive strips, diamond rotary instruments, and polishing pastes. These are available as one step and multistep polishing systems [16,17,18].

Flexible aluminum-oxide-coated, single-use discs such as Sof-Lex (3M) or super snap (Shofu) work in a dry field (these are manufacturers’ guidelines, although many dentists work with discs in a wet environment) and are used to polish smooth surfaces. Silicone discs (Enhance, Dentsply-Sirona) are pre-mounted, single-use, aluminum-oxide-impregnated, cured urethane dimethacrylate resin finishers designed for preparing composite surfaces for their final polish. They are used in a dry field as well, and do not remove additional material.

Tungsten carbide burs are a cutting instrument with geometrically defined blades [19]. The number of blades may vary between 10 to 30 blades. The tungsten carbide burs produce a smooth finish surface before polishing [20], are reusable, work in a wet field, and are mounted on high-speed air turbines.

Diamond burs are mounted on high-speed turbines and work in a wet field. The bur is coated with diamond grains. The smaller the particles, the higher the finish and polish ability. Opposed to tungsten carbide burs, diamond burs are abrasive instruments, not cutting instruments. The Shine 1–2 composite (Strauss & Co., Raanana, Israel) finishing kit contains three pairs of diamond burs; each pair has two roughness levels: 15 µ particle size (named xx-fine and marked purple) and <5 µ (named xxx-fine and marked white).

In the concurrent scientific literature, there is limited evidence regarding what polishing system is the best to achieve highly polished resin composite restorations. Many in vitro studies tried to compare different polishing systems. One of the greatest challenges in such studies is to isolate the tested parameter and eliminate confounding variables such as the pressure and angle put on the sample, the number of strokes, and ensuring a linear trajectory.

The present in vitro study compared two composite resin finishing and polishing systems: aluminum-oxide-coated Sof-Lex discs (3M ESPE, Seefeld, Germany), and a new diamond-particles-coated burs, system: Shine 1–2 (Strauss & Co., Raanana, Israel). The study used a custom-made device where the composite samples and rotary instruments were mounted on, thus simulating identical conditions for each sample and performing each test with the same angle and pressure, thus eliminating disruptive factors.

The purpose of the study was to compare the surface roughness and bacterial adherence to a composite treated by the two polishing systems. The surface roughness was evaluated using a Contour GT-K1 optical profilometer (Bruker, Billerica, MA, USA). The bacterial adherence was evaluated using crystal violet (CV) staining to measure the bacterial mass and bacterial count (CFU/mL), evaluating viable bacterial counts. The null hypotheses were that (1) there is no difference in the surface roughness of the three groups of samples: composites treated by the two polishing systems and the control group; and (2) there is no difference in bacterial adherence to the composite in the three groups of samples: composites treated by the two polishing systems and the control group.

## 2. Materials and Methods

### 2.1. Sample Preparation

Composite resin disc samples were fabricated using a Teflon template (20 wells, 5 mm height, and 8 mm diameter). Resin composite (G-aenial anterior, GC Europe, Leuven, Belgium) was packed into the wells, covered with a Mylar strip, and light-cured with a light curing device (D-Light Pro, GC Europe Leuven, Belgium) with a wavelength of 440–470 nm on high-power mode (1400 mW/cm^2^), according to the manufacturers’ instructions.

Ninety samples were divided to 3 groups (*n* = 30 samples each): a diamond bur composite finishing kit (Shine 1–2, Strauss & Co., Raanana, Israel), paper discs (Sof-Lex 3M ESPE, Seefeld, Germany), and a control group (C).

**Figure 1:**
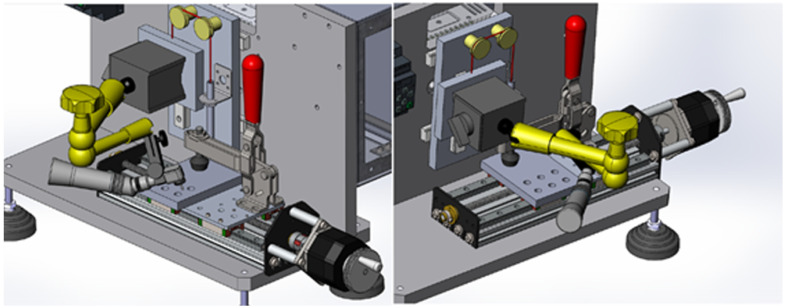
A custom-made device planned and fabricated for the test. A mechanical arm (yellow) holds the rotatory device in a fixed position. The samples are fixed on a tray by a lever (red). The tray moves on a fixed track, ensuring a similar pressure, angle, and number of strokes for every sample.

A standardized polishing process including the following variables was developed: the number of polishing strokes, the angle of the diamond bur/polish disc against the substrate, and the pressure implied on the substrate. For this purpose, a designated device was planned and constructed by Strauss & Co. laboratories (Figure 1). The device was composed of a mechanical arm and a mobile tray. The hand piece (a high-speed turbine for the diamond bur kit or a low-speed hand piece for the paper disc system) was mounted on the mechanical arm. The composite resin samples were mounted on the mobile tray. The tray moved along a fixed track at a fixed speed, 8 times back and forth. Each sample was polished with a constant number of strokes, in the same angle and with the same pressure exerted on it by the diamonds or discs, thus eliminating confounding variables.

Each group was treated by the company’s protocol:

The diamond xx-f bur was attached on a high-speed turbine parallel to the sample. After attaching the diamond bur, the turbine was operated at full speed (~400,000 RPM), and the tray’s device was turned on. The tray moved 8 times back and forth at a constant speed (~1.36 cm/s) against the diamond bur. Then, the xx-f bur was removed and the smoother xxx-f diamond bur was mounted on the turbine without changing its position and passed over the sample again.

The paper disc group was treated in the same manner using a low-speed hand piece (~20,000 RPM). The order of the discs was by the company’s protocol: black, dark blue, blue, and light blue. As with the diamond bur group, while changing the discs, the hand piece’s position stayed affixed.

Each group was tested for surface morphology quality using an optical profilometer (*n* = 10). The polished surface morphology effect on bacterial growth was also evaluated and included viable counts (colony forming unit CFU/mL) (*n* = 10) and bacterial biomass evaluation (crystal violet test) (*n* = 10).

### 2.2. Measuring the Effect of Different Polishing Systems on Sample Roughness

An optical profilometer (Countour GT-K1, Bruker) using an illumination wavelength was used for surface morphology scanning. Complex topographies were depicted on a micron scale. Each sample was scanned with a 5× magnification lens and an additional 0.55× optical magnification.

RA (surface roughness recordings taken from the average height of a profile above and below a center line) readings were taken from each sample.

### 2.3. Measuring the Effect of Different Polishing Systems on Bacterial Growth

*Streptococcus mutans* (UA-159) was used as test bacteria. Bacteria were cultured aerobically overnight at 37 °C in 5 mL of brain–heart infusion broth (BHI) (Difco, Detroit, MI, USA).

The top 10% of the suspension was harvested into a fresh test tube containing 5 mL of BHI and cultured overnight again. After culturing, the suspension was centrifuged for 15 min at 4000 rpm and adjusted to (OD = 1~108 CFU/mL).

A 0.5 mL drop of bacterial suspension was placed on the surface of each resin composite disc which was then placed in 10 mL of BHI broth and incubated at 37 °C for 48 h to allow the formation of 2-day-old biofilm.

Following 48 h of incubation, the discs were transferred into a new test tube with 2 mL of PBS followed by an ultrasonic bath for 15 min. A viable count evaluation (CFU/mL) of the bacteria attached to the discs was preformed (*n* = 10).

Bacterial biomass using crystal violet staining was also evaluated (*n* = 10). Discs were incubated for 48 h similarly as described above for bacterial viable count evaluation. Then, each disc was transferred to a 24-well plate and 1 mL of 100% methanol was added into each well for 20 min. The methanol was then collected without touching the samples or the well’s walls. One milliliter of 1% CV solution was added into each well for 20 min, and then excess CV solution was cleared and the discs were washed gently with DDW. One mL of 100% ethanol was added to dissolve the stained dye, and was vortexed with a pipettor for about 30 s. Two-hundred microliters of the stained ethanol were collected from the wells into a 96-well plate, and the optical density (OD) of the samples was determined by end-point measurement using the UV–VIS spectrophotometer at a wavelength of 538 nm.

### 2.4. Statistical Analysis

The data were analyzed by the Student’s *t* test to compare the means between the two groups, and one-way ANOVA between all groups. The level of significance was determined as *p* < 0.05. Statistical analysis was done using IBM SPSS Statistics 20 softwar (Chicago, IL, USA).

## 3. Results

### 3.1. The Effect of Different Polishing Systems on Sample Roughness

**Figure 2:**
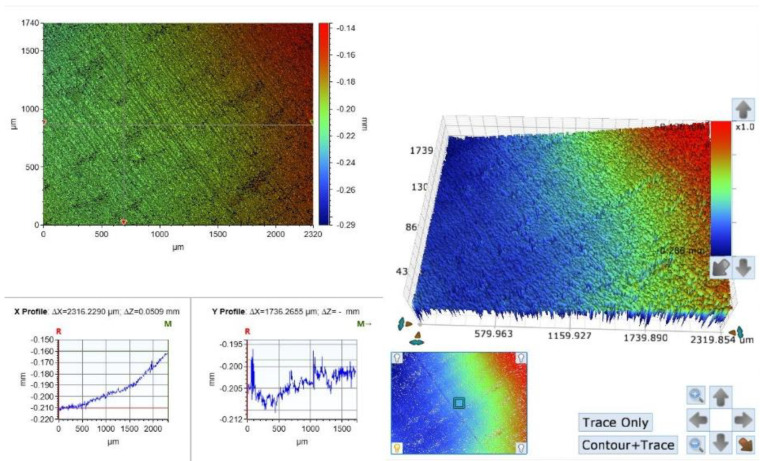
A surface scan photograph and a topography graph of a diamond burs group sample. Notice the homogenous parallel lines in the photo.

The first group examined was the diamond burs group. As can be observed in the optical photographs, polishing with the diamond burs created parallel lines carved on the surface with accordance to the bur’s shape, but the pattern is regular and homogenous along the sample (Figure 2). Surface RA values measured in this group ranged between 97 µ and 228 µ, with an average value of 169.4 µ (SD 45.2 µ) (Figure 2, Table 1).

**Figure 3:**
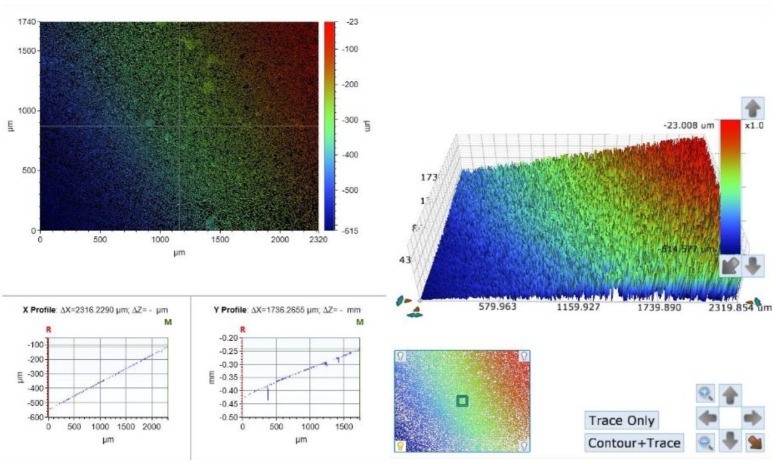
A surface scan photograph and a topography graph of a polishing discs group sample. An evenly polished surface with a homogenous grain pattern.

In the polishing discs group, the optical photographs present an evenly polished surface, but also a typical and homogenous grain all through the surface, in accordance with the coarse surface of the polishing discs (Figure 3). The surface RA values measured in this group were increased compared to the diamond bur group, ranging between 262 µ and 476 µ, with an average value of 364 µ (SD 77.7µ) (Figure 3, Table 1).

**Figure 4:**
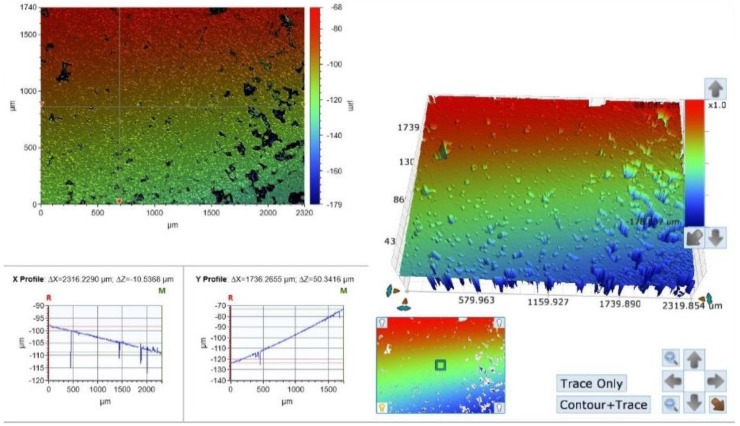
A surface scan photograph and a topography graph of a control group sample. Great variations were found between the different samples: smooth areas with occasionally high hills and deep pits.

In the control group, as can be seen in the optical photographs, the celluloid matrix provided very smooth areas, but simultaneously, some areas are characterized by irregular hills and deep pits (Figure 4). The surface RA values measured in this group were the lowest among the three groups tested, ranging between 102 µ and 154 µ, with an average value of 121.2 µ (SD 18.1 µ) (Figure 4, Table 1).

The photographs demonstrate a very irregular surface, and it is difficult to detect a non-random pattern on the sample surface. Moreover, the surface topography varies greatly between the different samples.

**Figure 5:**
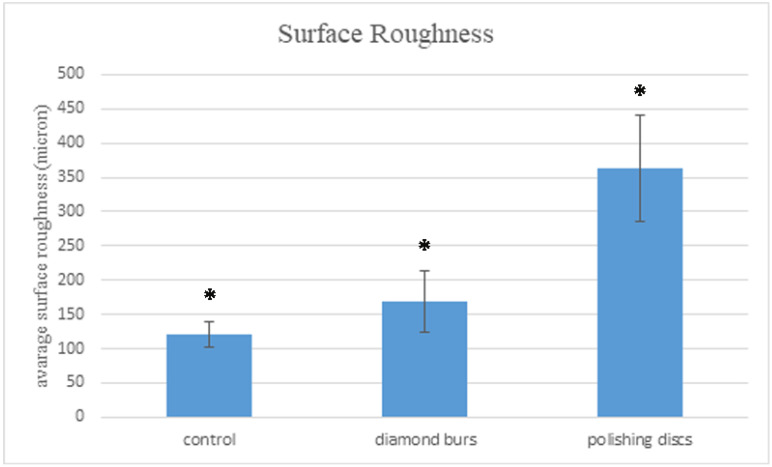
A surface RA comparison between the three test groups. A statistically significant difference was observed between the groups (* *p* < 0.00001).

A statistical analysis was performed between the three examined groups for the surface RA and standard deviation for each group. There was a significant difference between the groups (*p* < 0.00001). The results are distinct for *p* < 0.5, which are presented in Table 1 and Figure 5.

### 3.2. The Effect of Different Polishing Systems on Biofilm Mass

**Figure 6:**
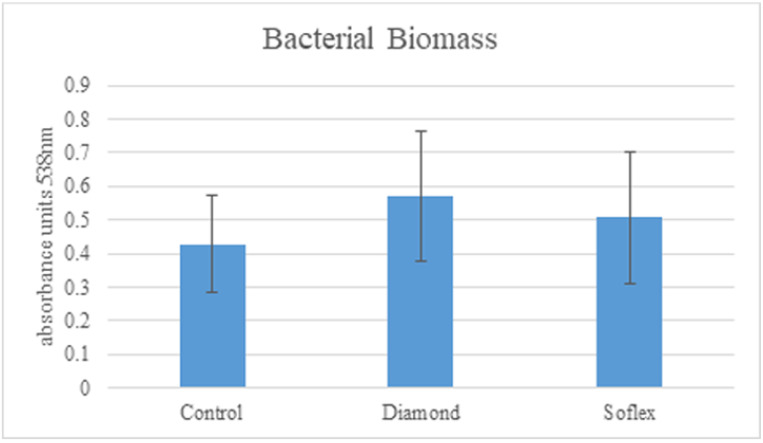
Biofilm biomass quantification using crystal violet staining after 48 h growth of *S. mutans* on discs polished by Shine 1–2 diamond burs, Sof-Lex discs, and non-polished discs that served as the control. The statistical significance in comparison with the control is *p* < 0.05.

The biomasses of *S. mutans* bacteria on the samples polished by the Shine 1–2 diamond burs were 0.458 ± 0.161, of the Sof-Lex discs were 0.507 ± 0.139, and of the control discs were 0.446 ± 0.142 (absorbance measured at 538 nm). Differences were not found to be statistically significant (*p* = 0.257) (Figure 6).

### 3.3. The Effect of Different Polishing Systems on Bacterial Growth

The effect of the two polishing systems on viable bacterial growth was evaluated using bacterial counts (CFU/mL) and compared to the control group. The viable cell count on the Shine 1–2 group was 2.25 × 10^4^ CFU/mL and on the Sof-Lex was 2.95 × 10^4^ CFU/mL. The viable cell counts on the control group were 2.75 × 10^4^ CFU/mL. (Figure 6).

No difference was observed between the two groups and the control (*p*-value = 0.856).

## 4. Discussion

A high-polished surface is a crucial condition for the success of dental restorations. Inappropriate finishing and polishing may result in plaque retention, secondary caries, gingival inflammation, and failure of the restoration. Other reasons for degradation of dental restorations are the harsh conditions in the oral environment such as parafunctional forces and erosive agents [4,5]. In this study, we compared two polishing systems for resin composite materials: diamond-particle-coated burs and paper discs coated with aluminum-oxide. A third group was evaluated as a control group: composite samples packed and cured as the test groups, and untouched by any polishing system.

The average surface roughness of the resin composite samples polished by the diamond-coated burs was statistically significantly lower than the samples polished by discs (169.4 µ, 364 µ, respectively). The surface roughness of the composite resin control group that was not polished at all was the lowest (121.2 µ) compared to the two test groups with a statistical significance. Biofilm biomass quantification and bacterial viable count showed no statistically significant differences between the three groups. The null hypothesis was partly confirmed, the results showing that there was a statistical difference in the surface roughness of composites treated by the two polishing systems, but without a difference in bacterial adherence to the composite.

The finishing and polishing of resin composite restorations involve manual dexterity and is highly technique-sensitive. Clinicians adapt different methods while polishing restorations in a patient’s mouth. The variables involved in this procedure are countless. In addition to the choice of which polishing agent to use—diamond burs, paper discs, tungsten carbide burs, etc.—other factors are involved in the process such as the number of strokes, the angle and the pressure exerted on the restoration, and the trajectory of the stroke. In most of the former studies evaluating and comparing different finishing and polishing systems in vitro, the polish was done by a clinician using a rotatory device held in hand and stroking over the restoration in a manner trying to simulate the clinical situation. This technique, used in a clinical trial, may introduce inherent errors and influence the outcome of the study.

For this purpose, an innovative device was planned and custom-made specifically for the study. The aim of the device was to eliminate the confounding variables and calibrate the process of the finishing and polishing of the samples for the test. The device ensured that every sample was treated similarly: the same number of strokes, identical angle and pressure, and a constant linear trajectory, thus avoiding confounding variables.

Two polishing systems were evaluated and compared in this study: Shine 1–2 composite (Strauss & Co. Raanana, Israel) and Sof-Lex (3M ESPE, Seefeld, Germany). The two systems share the same principle of using an abrasive agent applied sequentially in progressively finer grits, but have different features as well: the diamond bur system works in a wet field with high-speed RPM, is reusable and non-disposable, and requires lesser stages of work—two exchanges of diamond burs compared to four exchanges of discs. Polishing discs, on the other hand, are more flexible and adapt their shape to the restoration while stroking it, a significant attribute, especially when polishing class 3 and 4 restorations in anterior teeth.

Three different parameters were evaluated for each group in order to compare the effectiveness of the finish and polishing systems: surface RA, bacterial biomass, and bacterial viable load. The results of this study show that the highest polished surface is obtained by a Mylar strip placed on the uncured composite resin and then cured with UV light (Table 1, Figure 5). This treatment was used in this study as the control group. The control group also showed less bacterial biomass compared to the two tested groups (Figure 6). This result is not surprising, since a Mylar strip is a very smooth material, and placing it on the uncured and soft composite causes the composite to acquire the Mylar’s texture features. However, this technique is clinically non-applicable, since the morphology of occlusal posterior teeth does not permit its design to use a Mylar strip. Even when a Mylar strip is used for a class 3 restoration in an anterior tooth that has relatively smooth contours, there is still a need for finishing and polishing using an abrasive agent.

Surface morphology was tested by an optical profilometer with a 5× magnification lens and an additional 0.55× optical magnification. In the comparison of the surface RA test, the diamond bur group showed superiority over the discs group (Table 1, Figure 5). A statistically significant difference was observed between the groups.

The diamond burs group profilometer recording demonstrated a homogenous surface with regular parallel lines through all the samples (Figure 2). This is probably due to the geometrical shape of the bur used in the test (E5 flame shape). This bur is used to finish and polish smooth areas such as the buccal walls in class 5 restorations, and shape the cusps of posterior teeth to reach correct occlusal anatomy. The protocol of the test limited the direction of the bur upon the sample to one fixed trajectory, hence the parallel lines.

In the Sof-Lex discs group, the photographs display a homogenous surface, but not a smooth one, due to multiple microscopic pits created during the polishing process by the coarse surface of the polishing discs (Figure 3). This fact reflects in the high roughness value in this group. The writers’ opinion is that the limit of the direction of the paper discs’ contact against the sample, as was the case in the current study, does not influence the surface properties as dramatically as in the diamond bur group because of the inherent nature of the motion of the clinician using the discs to finish and polish: the paper disc is soft and adjusts itself to the surface, even if the hand piece is not parallel to the surface being treated.

Two methods were used to assess the efficiency of each polishing system in preventing adherence of bacteria to the polished surface. These two methods, i.e., viable count assay and CV staining, complement each other, allowing a reliable method to evaluate the viability and mass of bacteria on each group. The comparison of bacterial biomass (Figure 6) showed the highest levels in the diamond group, followed by the discs group, and the lowest in the control group, with no statistically significant difference. The bacterial viable count showed lowest counts in the diamond group followed by the control group, and highest in the discs group, again with no statistically significant difference. These results are not consistent with the results of the surface roughness, and it may imply that other factors are involved in the adherence of bacteria to a resin composite.

In addition to the pursuit of a means to achieve a smoother surface of resin composite restoration, the clinician also bears in mind the economical and time worthiness of the finishing and polishing system. The Shine 1–2 consists of a two-step finishing and polishing system, compared to the Sof-Lex system’s four steps. Additionally, diamond burs are reusable, compared to the single-use paper discs. On the other hand, this economical point of view may be hazardous, since re-use of the diamond burs in the event of an improper disinfection and sterilization process may be a threat to the patient.

## 5. Conclusions

This paper described a comparison between two different methods of finishing and polishing resin composite materials used in plastic dental restorations: diamond burs and paper discs. The two methods were compared to a control group as well. The finishing and polishing of the samples were done by a mechanical device with a constant angle, pressure, and number of strokes of the abrasive agent against the resin composite samples, thus standardizing the test.

The parameters tested were surface roughness (RA) and bacterial adherence (CFU/mL).

Within the limitations of this in vitro study, it may be concluded that both polishing methods influenced the surface roughness of tested resin composites.

The diamond bur kit produced significantly smoother surfaces compared to the paper disc kit.

No significant correlation was found between surface roughness and bacterial adherence.

These results can influence the choice of the finishing and polishing agents used by the dental practitioner after considering the resin composite restoration’s location, topography, and accessibility. The results achieved in this research indicate that further investigations are needed in this field, using higher resolutions and a combination of different finishing and polishing agents.

## Figures and Tables

**Table 1:** Surface RA values of tested groups.

	Control	Diamond Burs	Polishing Discs
N	10	10	10
Average value (micron)	121.2	169.4	364.0
Standard deviation (micron)	18.1	45.2	77.7

## Data Availability

Not applicable.
